# Narrow-band power dividers with wide range tunable power-dividing ratio

**DOI:** 10.1038/s41598-022-22178-0

**Published:** 2022-10-17

**Authors:** Sepehr Zarghami, Mohsen Hayati

**Affiliations:** Electrical Engineering Department, Faculty of Engineering, Razi University, Kermanshah, 67149-67346 Iran

**Keywords:** Electrical and electronic engineering, Engineering

## Abstract

This paper presents two narrow-band power dividers with a wide range power-dividing ratio based on the two new controlling insertion loss methods, which are low-impedance line and coupling capacitor. Initially, a narrow-band BPF is designed based on the equivalent circuit model and LC equivalent circuit. Then, using the surface current density, it is determined by which part of BPF structure the insertion loss (IL) can be controlled at center frequency. The tunable Wilkinson power dividers (TWPDs) are designed based on IL control components to create a wide range of power-dividing ratios, using only two DC voltages. The center frequency of first designed TWPD is 2.5 GHz, and the power-dividing ratio can be controlled up to 1:45 by variation of two DC voltages from 0 to 8 V. Since the structure of TWPDs are symmetric, the inverse voltages results in the inverted divided power between the output ports. The center frequency of second designed TWPD is 2.52 GHz, and power-dividing ratio can be controlled up to 1:134 by variation of two DC voltages from 1.7 to 4 V. Two proposed TWPDs are fabricated and measured. Comparisons of the measured and simulated results are presented to verify the theoretical predictions.

## Introduction

In most telecommunication systems, power dividers (PD) with equal and unequal power splitting ratios are as an essential element in the feeding network for the antenna array. Recently, the design of microwave circuits based on new methods such as slow wave applications of electromagnetically induced transparency in microstrip resonator^1^ and the automated framework for optimization of miniaturized microwave components^2^ have been considered by designers. Besides, in microwave wireless communication systems such as phased arrays and beam-steering networks, it is required that the signal divide as equal or unequal using power divider. The Wilkinson power dividers (WPD) are extensively used, with characteristics including: flexible power-dividing ratio, ability to remove DC, second and third harmonics, compact size, proper insertion and return losses, and good isolation between output ports.

To date, various types of WPD have been designed: The first category is PDs based on elliptic resonators and lowpass filters^3,4^, which are very good at suppressing harmonics up to high-order harmonics. But they are incapable of eliminating DC harmonics. The second category is the PDs based on the narrow-band^5–7^, dual-band^8^ and wide-band BPF^9–11^. Unequal WPD (UWPD) are another category for unequal signal division^12–21^. In^12,13^, the signal is divided into 1:2 ratio, but occupies very large size. In^13^, a structure using electromagnetic bandgap as a high-impedance transmission line (TL) is adopted for designing of UWPD. The power-dividing ratio is 1:3 in wideband, but return loss values are not proper in the working band. The UWPD with 1:4 power-dividing ratio^14,15^, which uses simple microstrip lines with different impedances, suffers from large size and inability to suppress unwanted harmonics. A 1:5 UWPD is designed in^16^ based on the offset double-sided parallel-strip lines. In^17,18^, a 1:6 unequal Gysel PD and a 1:10 UWPD are presented, respectively. One major disadvantage of^16–18^ is large size. Because designs^19–21^ use microstrip lines with unequal impedances without any resonator, they are suffering from large total size. In the mentioned PDs, it is not possible to consider any value of power-dividing ratio. To solve this problem, a Wilkinson and Gysel PDs with arbitrary power-dividing ratio are presented in^22–26^, respectively. In these PD type, the power division ratio can be adjusted arbitrarily during design process and before fabrication. As a result, the power-dividing ratio can no longer be changed after the design and fabrication processes.

Tunable power dividers (TPD) are another category that controls the operating band or power-dividing ratio, using a circuit consisting of lumped elements and DC voltages. The lumped elements circuit include DC-block capacitor, AC-block inductor, varactor diode and bias resistor. The DC voltage controls capacitance of varactor diode, therefore, the bandwidth or power-dividing ratio can be changed. In^27^ a 1:2 unequal TPD is presented based on the high-impedance TLs and four DC voltages. Some TPDs are presented in^28–31^. The power-dividing ratios are 1:1–1:2.4^28^, 1:13–1:28^29^, 1:2–1:100^30^ and up to 1:100^31^. The TPDs in^32,33^ controls both operating band and power-dividing ratio. The TPD of^32^ has a complex structure, as it uses 4 DC voltage sources and a large number of lumped elements. These disadvantages have been seen in TPD of^33^, which using ten individual DC voltages.

Among the designs examined, power-dividing ratio is an important feature, which has received less attention. Also arbitrary power-dividing ratio by a circuit, or a power divider that has a wide range of power-dividing ratio (whether equal or unequal) are not seen. As a result, there is a motivation to design and fabricate a PD that can adjust wide range of power-dividing ratio. Also, it has a simple design, and is more efficient in terms of the number of lumped elements and the DC voltage sources. The power dividers with tunable power-dividing ratio were employed in the design of antennas with polarization^34,35^ and pattern reconfiguration^36^. Besides, in many cases, unequal output powers at different output ports are required for systems like phased arrays and beam-steering networks^32^. It is also noted that all these applications requires narrow band power dividers with tuning power-dividing ratio.

In this paper, to resolve the aforementioned issues, two TPDs with wide range power-dividing ratio are designed, fabricated and measured. At first, a narrow-band BPF is designed based on the equivalent circuit model and LC equivalent circuit. Then using the surface current density, a resonator is specified, to which the lumped element circuit can be connected, and a PD is designed based on the proposed BPF. Next, two TPDs are designed based on the proposed PD and the lumped element circuits. Control of power-dividing ratios for fabricated TPDs are investigated and measured in the last section.

## Bandpass filter design

At first, a BPF with novel structure is analyzed and designed. Important parts of the proposed BPF structure are analyzed to determine which part is related to IL control. In this regard, the proposed BPF is fabricated and measured to confirm the results.

### Equivalent circuit model analysis

**Figure 1:**
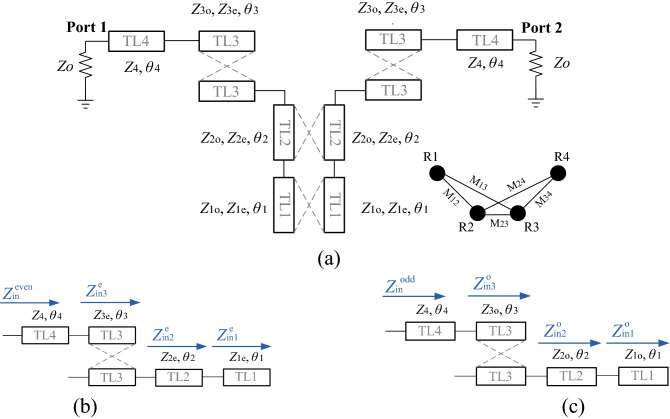
(**a**) Equivalent circuit model of the proposed BPF with coupling graph, (**b**) even and, (**c**) odd modes.

Figure 1a shows the equivalent circuit model of proposed BPF. The equivalent circuit model is based on the characteristics impedance (${Z}_{i}$) and electrical length (${\theta }_{i}$) of TLs, while equivalent circuit model has symmetric structure. The equivalent circuit model consists of two low-impedance lines with coupling effects (TL1), two high-impedance lines with coupling effect (TL2), two pair high-impedance lines with coupling effect and unequal impedances (TL3) and two high-impedance lines without coupling effect (TL4). Equivalent circuit model even and odd modes of the BP filter are shown in Fig. 1b, c, respectively. The even–odd mode analysis has been done step by step as below:

1 $$ Z_{{{\text{in}}1}}^{e} = \frac{{Z_{1e} }}{{j {\text{tan}} \theta_{1} }} = \frac{{ - Zj{\text{ cot}}2\theta }}{10} $$

2 $$ Z_{{{\text{in}}2}}^{e} = Z_{2e} \frac{{Z_{{{\text{in}}1}}^{e} + jZ_{2e} {\text{tan}} \theta_{2} }}{{Z_{2e} + jZ_{{{\text{in}}1}}^{e} {\text{tan}} \theta_{2} }} = \frac{{jZ\left( {{ }21{\text{ tan}}\theta - {\text{cot}}\theta } \right)}}{{21 - {\text{ tan}}^{2} \theta }} $$

3 $$ \left\{ {\begin{array}{*{20}l} {Z_{{{\text{in}}3}}^{e} = Z_{{{\text{x}}1}} \frac{{Z_{{{\text{in}}2}}^{e} + jZ_{{{\text{x}}1}} {\text{tan}} \theta_{3} }}{{Z_{{{\text{x}}1}} + jZ_{{{\text{in}}2}}^{e} {\text{tan}} \theta_{3} }} = \frac{{3A Z {\text{cot}}^{2} 3\theta }}{{60 {\text{cot}}^{2} 3\theta - 40 j {\text{tan}}\theta + B {\text{cot}}2\theta }}} \hfill \\ {Z_{{{\text{x}}1}} = 1/Y_{{{\text{x}}1}} } \hfill \\ {Y_{{{\text{x}}1}} = \frac{2j}{{ {\text{cot}} \theta_{3} \left( {Z_{3e} + Z_{3o} } \right)}} = \frac{{j2{\text{ tan}}3\theta }}{3Z}} \hfill \\ \end{array} } \right. $$

4 $$ Z_{{{\text{in}}}}^{{{\text{even}}}} = Z_{4} \frac{{Z_{{{\text{in}}3}}^{e} + jZ_{4} {\text{tan}} \theta_{4} }}{{Z_{4} + jZ_{{{\text{in}}3}}^{e} {\text{tan}} \theta_{4} }} = \frac{{Z\left( {2j\left( { - 10 + 11{\text{cos}}2\theta } \right){\text{sec}}^{2} \theta + 3C {\text{cot}}^{2} 3\theta } \right)}}{{D {\text{cot}}2\theta + 10E}} $$

And for odd mode:

5 $$ Z_{{{\text{in}}1}}^{o} = \frac{{Z_{1o} }}{{j {\text{tan}} \theta_{1} }} = \frac{{ - Zj {\text{cot}}2\theta }}{10} $$

6 $$ Z_{{{\text{in}}2}}^{o} = Z_{2o} \frac{{Z_{{{\text{in}}1}}^{o} + jZ_{2o} {\text{tan}} \theta_{2} }}{{Z_{2o} + jZ_{{{\text{in}}1}}^{o} {\text{tan}} \theta_{2} }} = \frac{{jZ\left( {{ }21{\text{ tan}}\theta - {\text{cot}}\theta } \right)}}{{21 - {\text{ tan}}^{2} \theta }} $$

7 $$ \left\{ {\begin{array}{*{20}l} {Z_{{{\text{in}}3}}^{o} = Z_{{{\text{y}}1}} \frac{{Z_{{{\text{in}}2}}^{o} + jZ_{{{\text{y}}1}} {\text{tan}} \theta_{3} }}{{Z_{{{\text{y}}1}} + jZ_{{{\text{in}}2}}^{o} {\text{tan}} \theta_{3} }} = \frac{{\left( { - 16 + Z^{4} {\text{cot}}^{4} 3\theta } \right) F {\text{tan}}^{4} 3\theta }}{{2Z^{7} {\text{cot}}2\theta {\text{cot}}^{2} 3\theta \left( {2j + {\text{cot}}^{2} 3\theta {\text{tan}}\theta } \right) + 4Z^{3} G}}} \hfill \\ {Z_{y1} = Z_{x1} - \frac{1}{{Z_{x1} Y_{{{\text{y}}1}}^{2} }}} \hfill \\ {Y_{{{\text{y}}1}} = \frac{2j}{{ {\text{cot}} \theta_{3} \left( {Z_{3e} - Z_{3o} } \right)}} = \frac{{j2{\text{ tan}}3\theta }}{Z}} \hfill \\ \end{array} } \right. $$

8 $$ {\text{Z}}_{{{\text{in}}}}^{{{\text{odd}}}} = Z_{4} . \frac{{Z_{{{\text{in}}3}}^{o} + jZ_{4} {\text{tan}} \theta_{4} }}{{Z_{4} + jZ_{{{\text{in}}3}}^{o} {\text{tan}} \theta_{4} }} = \frac{{H - \frac{1}{8}Z^{9} {\text{cot}}^{8} 3\theta {\text{csc}}\theta {\text{sec}}^{3} \theta \left( { - 36 + 44 {\text{cos}}4\theta + 20j {\text{sin}}2\theta + 11j {\text{sin}}4\theta } \right) + jZ {\text{I}}}}{{8jZ^{4} {\text{cos}}\theta \left( {1 - 2{\text{cos}}2\theta } \right)^{4} {\text{csc}}^{4} 3\theta J - 0.25j Z^{8} {\text{csc}}^{5} \theta \left( {1 - 2{\text{cos}}2\theta } \right)^{8} {\text{csc}}^{8} 3\theta K + I}} $$

where, in (3)–(8), parameters (A–K) are calculated as:

9 $$ \left\{ \begin{gathered} A = 30 + 20j {\text{tan}}\theta + {\text{cot}}2\theta \left( { - 2j + 3 {\text{tan}}\theta } \right) \hfill \\ B = 4j + 6 {\text{cot}}^{2} 3\theta {\text{tan}}\theta \hfill \\ C = - 30j + {\text{cot}}2\theta \left( { - 2 - 3j {\text{tan}}\theta } \right) + 41 {\text{tan}}\theta - {\text{tan}}^{3} \theta \hfill \\ D = 4 + 3 {\text{cot}}^{2} 3\theta {\text{tan}}\theta \left( { - 4j + 3{\text{ tan}}\theta } \right) \hfill \\ E = - 4 {\text{tan}}\theta + {\text{cot}}^{2} 3\theta \left( { - 6j + 9 {\text{tan}}\theta + 6j {\text{tan}}^{2} \theta } \right) \hfill \\ F = 8\left( { - 21 + {\text{tan}}^{2} \theta } \right) + Z^{4} {\text{cot}}^{4} 3\theta \left( {10 + 20j {\text{tan}}\theta + {\text{cot}}2\theta \left( { - 2j + {\text{tan}}\theta } \right)} \right) \hfill \\ G = - 84 + 5Z^{4} {\text{cot}}^{4} 3\theta - 10j Z^{4} {\text{ cot}}^{2} 3\theta {\text{tan}}\theta + 4 {\text{tan}}^{2} \theta \hfill \\ H = \left( {4Z^{5} \left( {1 - 2{\text{cos}}2\theta } \right)^{4} {\text{cot}}\theta {\text{csc}}^{4} \theta \left( { - 18 + 22{\text{cos}}4\theta + 20j {\text{sin}}2\theta + 11j {\text{sin}}4\theta } \right)} \right)/\left( {1 + 2{\text{cos}}2\theta } \right)^{4} \hfill \\ I = 4Z^{8} {\text{cot}}^{6} 3\theta \left( {{\text{cot}}2\theta - 10 {\text{tan}}\theta } \right) - 128 {\text{tan}}\theta \left( { - 21 + {\text{tan}}^{2} \theta } \right) \hfill \\ J = 22{\text{cos}}\theta + 22{\text{cos}}3\theta + 9j {\text{sin}}\theta + 11j {\text{sin}}3\theta \hfill \\ K = 44{\text{cos}}\theta + 44{\text{cos}}3\theta + 9j {\text{sin}}\theta + 11j {\text{sin}}3\theta \hfill \\ \end{gathered} \right. $$

It is to be noted that the Eqs. (3) and (7), which are the equations of the coupled lines of TL3, are based on the input impedance of two-port Z-matrix coupled lines “$$\pi$$” model^37^. Based on the “$$\pi$$” model for coupled lines, the two port Z-matrix and its parameters taking into account the boundary conditions for TL3 coupling lines, i.e., $$I_{2} = I_{4} = 0, $$ are obtained as follows^38^:

10 $$ \left[ {\begin{array}{*{20}c} {V_{1} } \\ {V_{2} } \\ \end{array} } \right] = \left[ {\begin{array}{*{20}c} {\begin{array}{*{20}c} {Z_{11} } & {Z_{12} } \\ \end{array} } \\ {\begin{array}{*{20}c} {Z_{21} } & {Z_{22} } \\ \end{array} } \\ \end{array} } \right]\left[ {\begin{array}{*{20}c} {I_{1} } \\ {I_{2} } \\ \end{array} } \right] $$

where

11 $$ \left\{ {\begin{array}{*{20}l} {Z_{11} = Z_{22} = - j0.5\left( {Z_{e} + Z_{o} } \right){\text{cot}}\theta } \hfill \\ {Z_{12} = Z_{21} = - j0.5\left( {Z_{e} - Z_{o} } \right){\text{cot}}\theta } \hfill \\ \end{array} } \right. $$

Then, the input impedance ($Z_{in}$) can be calculated as follows:

12 $$ Z_{in} = Z_{11} - \frac{{Z_{12} Z_{21} }}{{Z_{22} + Z_{L} }} $$

In the following, for even and odd mode analysis, $Z_{L}$ is considered as two values of infinity and 0, respectively. As a result Eqs. (3) and (7) are calculated as follows::

13 $$ \left. {Z_{{{\text{in}},{\text{ even}}}} } \right|_{{Z_{L} = \infty }} = Z_{11} = 1/Y_{{{\text{x}}1}} $$

14 $$ \left. {Z_{{{\text{in}},{\text{ odd}}}} } \right|_{{Z_{L} = 0}} = Z_{11} - \frac{{Z_{12}^{2} }}{{Z_{11} }} = Z_{x1} - \frac{1}{{Z_{x1} Y_{{{\text{y}}1}}^{2} }} $$

**Figure 2:**
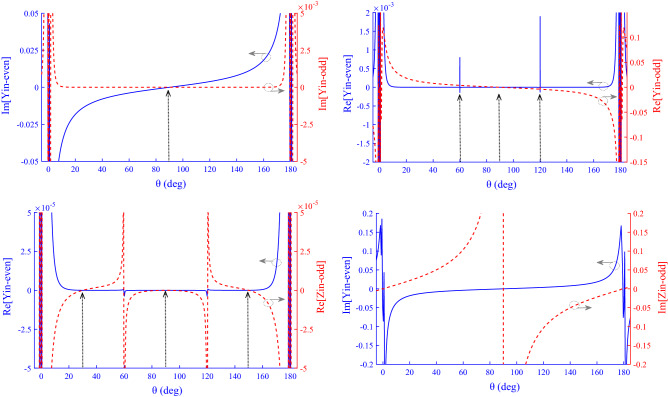
$${Y}_{in}^{even}$$, $${Y}_{in}^{odd}$$ and $${Z}_{in}^{odd}$$ curves.

The following equations are used to obtain important parameters of the structure:

15 $$ {\text{S}}_{21} = \frac{{\left( {Z_{{{\text{in}}}}^{{{\text{even}}}} - Z_{{{\text{in}}}}^{{{\text{odd}}}} } \right)Z_{0} }}{{\left( {Z_{{{\text{in}}}}^{{{\text{even}}}} + Z_{0} } \right) \cdot \left( {Z_{{{\text{in}}}}^{{{\text{odd}}}} + Z_{0} } \right)}} $$

16 $$ {\text{S}}_{11} = \frac{{Z_{{{\text{in}}}}^{{{\text{even}}}} Z_{{{\text{in}}}}^{{{\text{odd}}}} - Z_{0}^{2} }}{{\left( {Z_{{{\text{in}}}}^{{{\text{even}}}} + Z_{0} } \right) \cdot \left( {Z_{{{\text{in}}}}^{{{\text{odd}}}} + Z_{0} } \right)}} $$

where $Z_{0} = 50$ ohm. Based on the coupling graph (Fig. 1a) it is clear that the proposed equivalent circuit model has four resonators (R1–R4). For the proposed equivalent circuit model, the values of impedance and electrical length of the lines are considered as follows: $Z_{2o} = Z_{2e} = Z_{4} = Z{ }$ and $Z_{1o} = Z_{1e} = Z/10$. Also, asymmetric coupling lines (TL3) have unequal impedance and the impedance values are considered as $Z_{3e} = 2Z$ and $Z_{3o} = Z$. On the other hand, the electrical length of the lines is considered as: $\theta_{2} = \theta_{4} = \theta$ and $\theta_{1} = 2\theta$ and $\theta_{3} = 3\theta$. TL1s have low impedance and other lines have high impedance. Since the value of $Z$ for high-impedance and low-impedance lines can be considered 150 and 15 ohm^39^, respectively, so the impedance of TL1 is equal to $Z/10$. Accordingly, Eqs. (1)–(14) are calculated and simplified in Wolfram Mathematica software, then the $\mathcal{R}\mathcal{e}$ and $\mathcal{I}\mathcal{m}$ parts are separated, and each part is plotted in MATLAB according to $\theta$, as shown in Fig. 2. A proper narrow-band BPF has a center frequency (*f*_o_) with low IL, and has two TZs to have a good stopband bandwidths and sharp roll-off. Low IL value is obtained by creating a pole at *f*_o_, that according to (15) S_11_ = 0 or, ${\mathrm{Y}}_{\mathrm{in}}^{\mathrm{even}}\cdot {\mathrm{Y}}_{\mathrm{in}}^{\mathrm{odd}}=1/{{Z}_{0}}^{2}$.

Because ${Z}_{0}=50$ ohm, therefore, $\mathcal{R}\mathcal{e}[{\mathrm{Y}}_{\mathrm{in}}^{\mathrm{even}}]=(1/2500)\times \mathcal{R}\mathcal{e}[{\mathrm{Z}}_{\mathrm{in}}^{\mathrm{odd}}]$ and $\mathcal{I}\mathcal{m}[{\mathrm{Y}}_{\mathrm{in}}^{\mathrm{even}}]=(1/2500)\times \mathcal{I}\mathcal{m}[{\mathrm{Z}}_{\mathrm{in}}^{\mathrm{odd}}]$. For TZs according to (16), its need S_21_ = 0 or ${Z}_{in}^{odd}={Z}_{in}^{even}$. For ${Y}_{in}=1/{Z}_{in}$ then ${\mathrm{Y}}_{\mathrm{in}}^{\mathrm{even}}={\mathrm{Y}}_{\mathrm{in}}^{\mathrm{odd}}$, therefore, the resonant conditions are equal at the intersection points of $\mathcal{R}\mathcal{e}[{\mathrm{Y}}_{\mathrm{in}}^{\mathrm{even}}]$ with $\mathcal{R}\mathcal{e}[{\mathrm{Y}}_{\mathrm{in}}^{\mathrm{odd}}]$ and $\mathcal{I}\mathcal{m}[{Y}_{in}^{even}]$ with $\mathcal{I}\mathcal{m}[{Y}_{in}^{odd}]$. Figure 2 shows curves of ${\mathrm{Y}}_{\mathrm{in}}^{\mathrm{even}}$, ${\mathrm{Y}}_{\mathrm{in}}^{\mathrm{odd}}$ and ${\mathrm{Z}}_{\mathrm{in}}^{\mathrm{odd}}$ to determine the intersection point of the curves and the optimal value of $\theta$. As illustrated, a 90° has the best conditions. In other words, each resonator in the equivalent circuit model of Fig. 1a needs to have a length equal to $\theta =\lambda /4$.

### LC equivalent circuit

**Figure 3:**
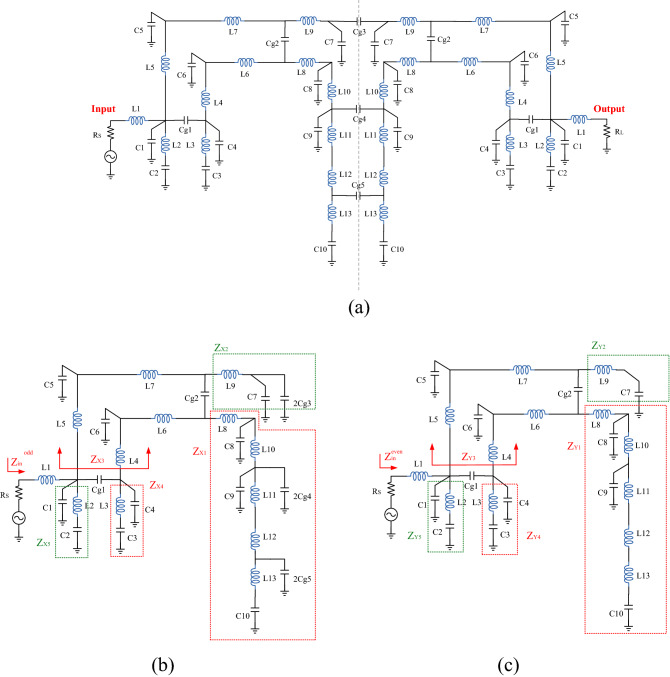
(**a**) LC equivalent circuit, (**b**) odd and, (**c**) even modes.

The LC equivalent circuit based on coupling capacitors (using capacitors instead of coupling effect) gives a view of what parts and couplings of the proposed BPF have an effect on IL at the center frequency. Adding varactor diode (variable capacitor) to high capacitive effect (wide area) and important couplings sections play the most important role in the design of power dividers with tunable power dividing ratio. Since the structure based on asymmetric coupling has design complexity, so for the proper response, it is necessary to optimize parameters of the equivalent circuit model. To identify the starting points of the optimization process, the LC equivalent circuit is considered, in the design path of the proposed BPF, as shown in the Fig. 3. In LC equivalent circuit, each TL replaced by one inductor (L_i_, where i = 1–13) and one capacitor (*C*_i_, where i = 1, 2,… 10), and a capacitor is considered for each coupling effect (*C*_gi_, where i = 1, 2, … 5). The inductor and capacitor value can be extracted from^4,40^. Due to LC equivalent circuit has the symmetry structure, even and odd mode analysis have been performed. Figure 3b, c shows the odd/even modes, respectively. The input impedance can be calculated step by step in both even and odd modes as follows:

17 $$ \left\{ {\begin{array}{*{20}l} {Z_{x1} = \left( {\left( {\left( {a\parallel \frac{1}{{s(C_{9} + 2C_{g4} )}}} \right) + sL_{10} } \right)\parallel \frac{1}{{sC_{8} }}} \right) + sL_{8} } \hfill \\ {a = b + s\left( {L_{11} + L_{12} } \right)} \hfill \\ {b = \left( {\frac{1}{{sC_{10} }} + sL_{13} } \right)\parallel \frac{1}{{2sC_{g5} }}} \hfill \\ \end{array} } \right. $$

18 $$ Z_{x2} = \frac{1}{{s(C_{7} + 2C_{g3} )}} + sL_{9} $$

19 $$ \left\{ {\begin{array}{*{20}l} {Z_{x3} = \left( {\left( {c + s\left( {L_{6} + L_{7} } \right)} \right)\parallel \frac{1}{{s(C_{5} + 2C_{6} )}}} \right) + s\left( {L_{4} + L_{5} } \right)} \hfill \\ {c = \left( {Z_{x1} + Z_{x2} } \right)\parallel \frac{1}{{sC_{g2} }}} \hfill \\ \end{array} } \right. $$

20 $$ Z_{x4} = \left( {\frac{1}{{sC_{3} }} + sL_{3} } \right)\parallel \frac{1}{{sC_{4} }} $$

21 $$ Z_{x5} = \left( {\frac{1}{{sC_{2} }} + sL_{2} } \right)\parallel \frac{1}{{sC_{1} }} $$

22 $$ \left\{ {\begin{array}{*{20}c} {Z_{{{\text{in}}}}^{{{\text{odd}}}} = \left( {d\parallel Z_{x5} } \right) + sL_{1} } \\ {d = \left( {Z_{x3} \parallel \frac{1}{{sC_{g1} }}} \right) + Z_{x4} } \\ \end{array} } \right. $$

And for even mode:

23 $$ \left\{ {\begin{array}{*{20}l} {Z_{Y1} = \left( {e\parallel \frac{1}{{sC_{8} }}} \right) + sL_{8} } \hfill \\ {e = \left( {f\parallel \frac{1}{{sC_{9} }}} \right) + sL_{10} } \hfill \\ {f = \frac{1}{{sC_{10} }} + s\left( {L_{11} + L_{12} + L_{13} } \right)} \hfill \\ \end{array} } \right. $$

24 $$ Z_{Y2} = \frac{1}{{sC_{7} }} + sL_{9} $$

25 $$ \left\{ {\begin{array}{*{20}l} {Z_{Y3} = \left( {\left( {g + s\left( {L_{6} + L_{7} } \right)} \right)\parallel \frac{1}{{s(C_{5} + 2C_{6} )}}} \right) + s\left( {L_{4} + L_{5} } \right)} \hfill \\ {g = \left( {Z_{Y1} + Z_{Y2} } \right)\parallel \frac{1}{{sC_{g2} }}} \hfill \\ \end{array} } \right. $$

26 $$ Z_{Y4} = \left( {\frac{1}{{sC_{3} }} + sL_{3} } \right)\parallel \frac{1}{{sC_{4} }} $$

27 $$ Z_{Y5} = \left( {\frac{1}{{sC_{2} }} + sL_{2} } \right)\parallel \frac{1}{{sC_{1} }} $$

28 $$ \left\{ {\begin{array}{*{20}l} {Z_{{{\text{in}}}}^{{{\text{even}}}} = \left( {h\parallel Z_{Y5} } \right) + sL_{1} } \hfill \\ {h = \left( {Z_{Y3} \parallel \frac{1}{{sC_{g1} }}} \right) + Z_{Y4} } \hfill \\ \end{array} } \right. $$

Finally, the resultant S-parameters are evaluated with (15) and (16). After calculating S_21_ based on the LC equivalent circuit, it has been determined that almost all of the proposed BPF structure is effective in controlling the location of the TZs. However, *L*_13_ and *C*_10_ have less effect. As a result, there is a general equation with several variables, to achieve the three goals of filter design. The goals of filter design are as follows: central frequency at 2.5 GHz (S_21_@ 2.5 GHz = 0 dB), first TZ located at 2.2 GHz (S_21_@ 2.2 GHz = − ∞ dB) and second TZ located at 2.8 GHz (S_21_@ 2.8 GHz = − ∞ dB). In this way, the optimization process has been used based on the gradient algorithms in simulation software of ADS to obtain goals of filter design.

### Layout design

**Figure 4:**
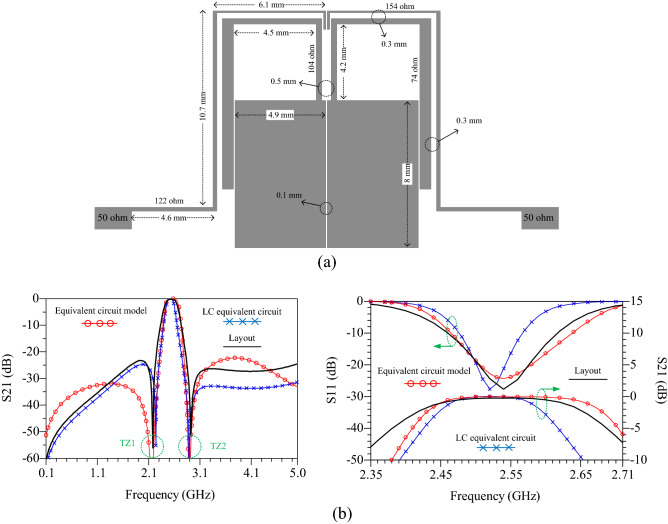
The proposed BPF, (**a**) layout, (**b**) simulation results S_21_ and S_11_ of equivalent circuit model, LC equivalent circuit and layout.

After the calculations, the proposed BPF is designed by optimizing parameters in the ADS software environment. The layout structure and simulated responses of the designed BPF are shown in Fig. 4a. Figure 4b shows simulation results of the proposed BP filter. The center frequency (*f*_o_) in the passband is 2.54 GHz and the − 3 dB (IL) bandwidth is 0.23 GHz, from 2.405 to 2.635 GHz. The insertion and return losses at *f*_o_ are 0.26 and 27.7 dB, respectively. These results show that the passband of BPF has a proper safety margin for frequency 2.5 GHz. It is also illustrated that the designed BPF has two TZs at frequencies of TZ1 = 2.18 and TZ2 = 2.92 GHz. The 0.74 GHz frequency gap between TZs shows sharp roll-off of this filter. The upper stopband bandwidth is extend to 2.2 *f*_o_, with 20 dB suppression level.

Figure 6a shows the IL at *f*_o_ versus variation of gap distance between TL1s and *C*_g5_ value. To extend the bandwidth, the gap distance between two TL1s should be less than 0.1 mm, as shown in Fig. 6b. To achieve this goal, fabrication costs increases, significantly. As a result, to reduce the fabrication cost, the minimum design resolution of 0.1 mm has been considered.

### Tuning of the insertion loss at center frequency

**Figure 5:**
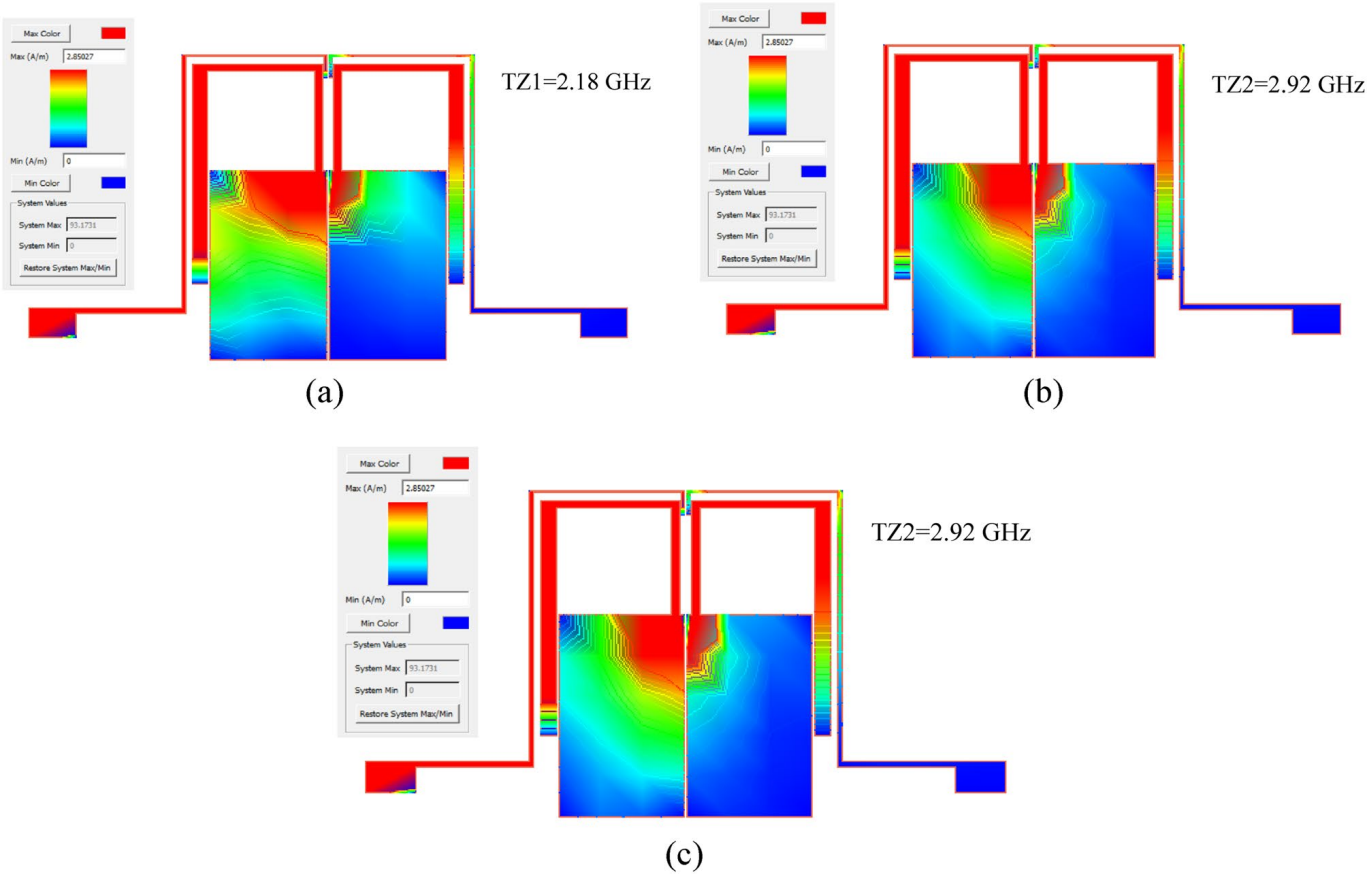
The surface current density at (**a**) TZ1, (**b**) TZ2 and (**c**) *f*_o_.

**Figure 6:**
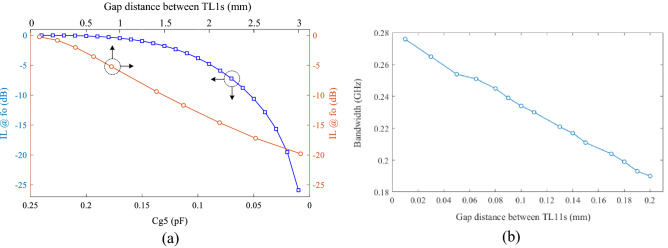
(**a**) The insertion loss value at *f*_o_ versus variation of gap distance between TL1s and Cg5 value, (**b**) variation of bandwidth versus gap distance between TL1s.

**Figure 7:**
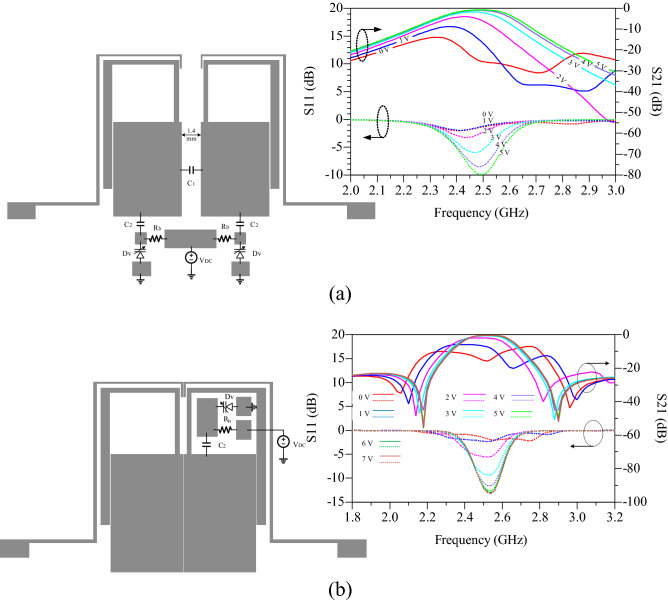
The proposed BPFs with control of passband IL, (**a**) proposed circuit and simulation response depending on V_DC_, (**b**) second design BPF, and simulation response depending on V_DC_.

This section identifies which parts of the proposed BPF structure have a direct effect on IL at the *f*_o_. As a result, the value of IL can be adjusted by adding a lumped element circuit containing the varactor diode (DC voltage controllable capacitance) to these parts. Figure 5 shows the surface current density of the proposed BPF at the TZs and *f*_o_. As shown in Fig. 5a, b, the parts, which are mostly red, have a greater effect on the TZs. Therefore, TZs location depend on TLs of TL2, TL3, TL4, TL5 and one of TL1s (TL1 that is close to the input port). In other words, TL1 on the output port side has very little effect on TZs. Figure 5c shows that the TL1 near the output port has a high effect on the central frequency of passband. Based on the surface current density simulations, the TL1 on the output port side plays a direct role for all frequencies of passband. Because TL1s are low impedance lines and plays the role of a capacitor with a high capacitance value, this stub can be controlled by setting its dimensions. On the other hand, the gap between two TL1 stubs (The dotted line in Fig. 3a), which are modeled in the proposed equivalent circuit with *C*_g3_, *C*_g43_ and *C*_g5_, has a direct effect on the IL at *f*_o_. In the optimization process, it is found that the effect of *C*_g5_ is greater. The lower the value of capacitor *C*_g5_, the lower the IL at *f*_o_, and vice versa. As results, the greater the gap between the two TL1 lines, the higher the IL value in the bandwidth. Figure 6 shows the IL at *f*_o_ versus variation of gap distance between TL1s and *C*_g5_ value. Since in the previous section, this gap was modeled by a capacitor, in the case of the maximum gap distance (S_21_(@ *f*_o_) = − 20 dB), it is possible to use an SMD capacitor between two TL1s. Then by adjusting the value of SMD capacitor, the value of IL in the passband can be controlled. Accordingly, a BPF with control of passband insertion loss is designed and simulated as shown in Fig. 7a. The lumped element circuit of Fig. 7a structure includes:

1. The varactor diode (Dv) is SMV1247-079LF with a capacitance of 0.64–8.86 pF within a range of DC voltage (V_DC_) variations 0–8 V.
2. Capacitors C_1_ and C_2_ are Murata GRM0603 with value of 6 and 1.5 pF, respectively.
3. R_b_ is the bias resistor with a value of 100 kΩ and V_DC_ is DC voltage with value range of 0–8 V.

The simulation response of this circuit is shown in Fig. 7a. The values of S_21_ at the frequency of 2.5 GHz are equal to − 7.25, − 8.16, − 6.2, − 2.1, − 1.14 and − 0.85 dB for voltages of 0, 1, 2, 3, 4 and 5 V, respectively. As mentioned in the previous section, both TL1s do not have the same effect on the IL at central frequency. The effect of TL1, which close to the output port, is dominant over the other TL1. As a result, second design BPF containing just elements connected to the left TL1 is designed and simulated, as shown in Fig. 7b. The simulated results show that value of IL at *f*_o_ is changed according to DC voltage, without elimination of TZs. Also fewer lumped elements are used. Because the value of signal passing through the proposed BPF is controlled by the voltage, the proposed BPFs can be used in the design of WPD with wide range tunable power-dividing ratios.

## Design of power divider

**Figure 8:**
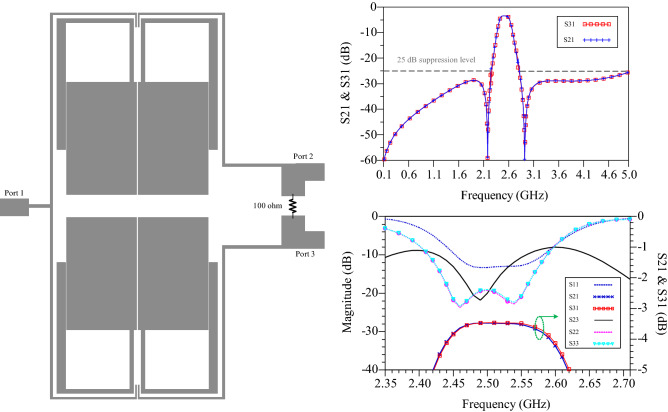
Narrow-band WPD proposed circuit and its S-parameter results.

Using designed BPF in the previous section, a narrow-band WPD is designed as shown in Fig. 8. The isolation resistor is 100 ohms. The simulation responses of narrow-band WPD the passband bandwidth is 0.14 GHz from 2.45 to 2.59 GHz with an attenuation level of − 3 dB. Simulation S_11_ in the passband is less than − 13.3 dB, S_22_ and S_33_ is less than − 19 dB as shown in Fig. 8. The isolation (S_32_) is less than − 20 dB at *f*_o_ = 2.5 GHz. The lower and upper stopbands have a high suppression level and suppress the harmonics of DC, second and third harmonics for the central frequency with a suppression level more than 25 dB.

## Control of power-dividing ratio

The section of tuning IL at *f*_o_ shows that TL1, which is near the output port, has a greater effect on the central frequency in the passband bandwidth. Also, the gap distance between two TL1s, increases or decreases the IL at the central frequency. Therefore, to design a PD with controlling the power-dividing ratio between the two output ports, it is possible to use the TL1 close to the output and the TL1s gap. Accordingly, two WPD designs have been reviewed by controlling the ratio of power-dividing in this section:

### Control of power-dividing ratios with the coupling capacitor

**Figure 9:**
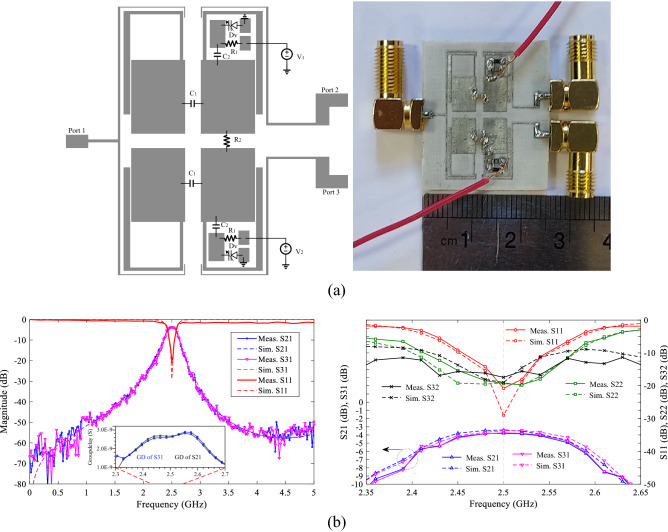
Proposed WPD with tunable power-dividing ratio (first design), (**a**) layout and photograph of fabrication structure, (**b**) simulation and measurement results at V1 and V2 = 8 V.

**Figure 10:**
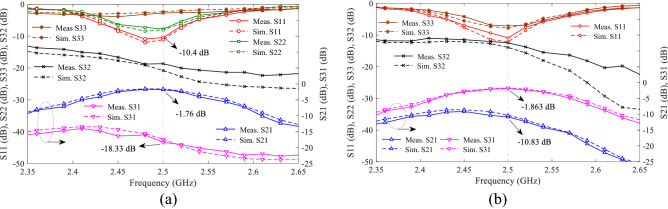
Measurement and simulation results of first design TWPD, (**a**) at V1 = 0 and V2 = 8, and (**b**) at V1 = 8 and V2 = 1.

In Fig. 7, the proposed BPF can control the IL in the passband by using a capacitor C_1_ between the TL1s and a capacitive control circuit including a varactor diode, bias resistor, auxiliary capacitor C_2_ and a DC voltage source. The responses showed that, although IL is controlled, the TZs are eliminated and the roll-off rate is reduced. But using the voltage range of 0–8, IL value can be changed between − 0.85 and − 25.7 dB at *f*_o_ = 2.5 GHz. By using this filter, a TWPD with control of IL is designed. Thus, proposed BPF are used instead of quarter wavelength lines in WPD^4^ as first design. The layout circuit, simulation and measurement results of the first design are shown in Fig. 9. The capacitor of C_1_ is GRM0115C murata 1.8 pF, capacitor of C_2_ is GRM0115C murata 1.5 pF, and bias resistors are considered 10 kohm. The varactor diodes are SMV1247 model and the values of DC voltages V1 and V2 changes in the range of 0–8 V. This PD is fabricated and measured as the first design, tunable power divider with control of power-dividing ratios using the gap distance in the line of symmetry. Figure 9a shows the layout of first design TWPD and photograph of fabrication, where R_2_ = 100 Ω is the isolation resistor, and can be considered when equal division of power is needed with high isolation. It should be noted that, for unequal dividing of power to the any desired value, isolation resistor may have a destructive effect. For example, in the presence of isolation resistor, by changing the voltages of V1 and V2, the divided power does not exceed − 3 dB. Therefore, this resistor can be eliminated to achieve a wide range of power divided ratio. On the other hand, adding resistor increases the matching and isolation at the output ports. Figure 9b shows the simulation and measurement results for both voltages V1 = V2 = 8 V, in essence equal division of power. The maximum variation of group delay (GD) in the passband for S_21_ and S_31_ are 0.65 and 0.63 ns, respectively. Figure 10a, b shows the unequal power divided for voltages V1 = 0, V2 = 8 and V1 = 8, V2 = 1, respectively. For this power divider, the larger the difference between the values of V1 and V2, the greater the difference in power-dividing ratio between the output ports. For example, if V1 = 8 and V2 = 0 V, which is the maximum different between the two DC voltages, the value of S_31_ = − 1.765 dB and S_21_ = − 18.33 dB. That is, the power ratio is 1:45 at a frequency of 2.5 GHz. Since the structure is symmetric, inverse voltages (V1 = 0, V2 = 8) results in the inverted power-dividing ratio between the output ports (S_31_ = − 18.91 dB, S_21_ = − 1.78 dB). As the DC voltage of a section decreases, the value of power-dividing to the output port of that section decreases. As example, two voltages of V1 = V2 = 0 results.

S_21_ = − 18.36 dB, S_31_ = − 18.93 dB. Scilicet, by selecting these two voltages, the entire power can be blocked at the central frequency.

### Control of power-dividing ratios with low-impedance lines

**Figure 11:**
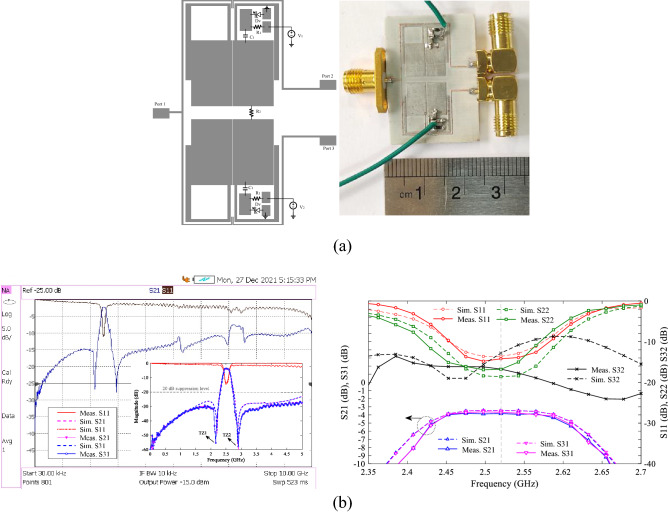
Proposed WPD with tunable power-dividing ratio (second design), (**a**) layout and photograph of fabrication structure, (**b**) simulation and measurement results at V1 and V2 = 4 V.

**Figure 12:**
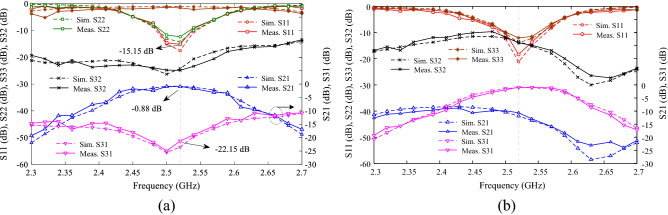
Measurement and simulation results of second design TWPD, (**a**) at V1 = 4 and V2 = 1.7, and (**b**) at V1 = 4 and V2 = 2.15.

In section of tuning IL at *f*_o_, it was shown that TL1 on the output port side has a direct effect on *f*_o_. As a result, by controlling the capacitance effect of TL1 stub using SMD capacitor and the varactor diode, the power-dividing ratio can be control for the design of a TWPD (second design). Figure 11a shows the layout circuit and fabrication photograph of the second design. This design does not have a symmetry line capacitor. As a result, two elements are reduced and the size become smaller. Because PD is controlled using the capacitance effect of TL1, there is a wider range to divide unequal power over a narrower range DC voltage. So that the DC voltage for equal to unequal power-dividing between 1.7 and 4 V are sufficient for DC voltage sources. The capacitor of C_1_ is GRM0115C murata 30pF, and bias resistors are considered 10 kohm. The varactor diodes are SMV1247 model and the values of DC voltages V1 and V2 changes in the range of 0–4 V. Figure 11b shows second design simulation and measurement results for V1 = V2 = 4 V. As shown, unlike the first design TWPD, the TZs are not eliminated in second design TWPD, resulting in a high sharp roll-off. The maximum variation of group delay (GD) in the passband for S_21_ and S_31_ are 0.85 and 0.64 ns, respectively. As shown in Fig. 11b, S_32_ is equal to − 11.34 dB, which is relatively proper value without R_2_. It can also be seen that the lower and upper stopbands are wide with a suppression level of 20 dB. The values of input matching (S_11_), output matchings (S_22_ and S_33_) and isolation between two output ports (S_23_) are appropriate in the 0.1 GHz bandwidth range, as shown in Fig. 11b. Figure 12a, b shows the unequal power divided for voltages of V1 = 1.7, V2 = 4 and V1 = 4, V2 = 2.15, respectively. For this PD, the farther the values V1 and V2 are from each other, the greater the difference in power divided. For example, if V1 = 4 and V2 = 1.7 V, which the values of S_31_ = − 0.877 and S_21_ = − 22.15 dB. That is, at these voltages, the power-dividing ratio is 1:134 at frequency of 2.52 GHz. The power-dividing ratio of.

**Figure 13:**
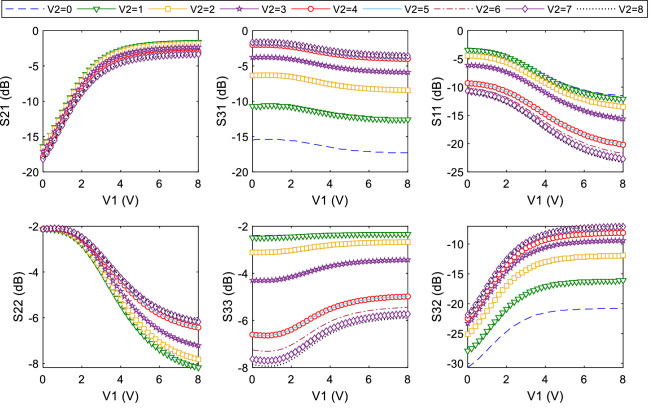
Wide range simulation S-parameters results of the proposed TWPD (first design).

1:134 is not seen in the first design. Since the structure of second TWPD is symmetrical, at opposite DC voltages (V1 = 1.7, V2 = 4 V) the value of divided power between the output ports is reversed (S_31_ = − 22.28, S_21_ = − 0.79). As a DC voltage decreases, the value of power transmitted to output port decreases. As example, for two voltages V1 = V2 = 1.7, results S_21_ = − 22.23 dB, S_31_ = − 22.31 dB. That is, by selecting these two voltages, the entire power can be blocked at *f*_o_. The advantage of the second is the maximum different value between the two DC voltages, design over the first design is more compact size, more compact variation ranges for DC voltages, and a wide divided power range up to 1:134. Figure 13 shows the variation of S-parameters for voltage variation V1 = 0 to 8 (with step 0.1 V) and V2 = 0 to 8 (with step 1 V). Using these graphs, the simulation responses of the S-parameters can be investigated for important power divided ratios. It is also possible to choose any power-dividing range from equal to unequal. The important ranges of divided power for the second design TWPD at constant voltage V2 = 4 and V1 = 4 are tabulated in Tables 1 and 2, respectively. According to these tables, it is clear that the divided power between the two output ports is done symmetrically by adjusting the voltages. Also, the larger the voltage difference between V1 and V2, the wider range of divided power continues. Table 3 compares the results of both proposed power dividers with previous works. It is easy to compare from Table 3 that the wide range of divided power, the number of low DC voltages, the range of low DC voltages and the compact size are the most important advantages of the two proposed designs. In Table 3, FPDR is fractional power-dividing ratio to evaluate the capability of a TWPD in the unequal power dividing, which can be derived as follows:

29 $$ {\text{FPDR}} = \frac{{\left( {{\text{MPDR}} - 1} \right) \times \left( {{\text{IL}}_{{{\text{max}}}} - {\text{IL}}_{{{\text{min}}}} } \right)}}{{{\text{MPDR}} \times {\text{IL}}_{{{\text{max}}}} }} \times 100 $$

**Table 1:** Important ranges of divided power for second design TWPD at V2 = 4.

Range	1:1	1.5:1	2:1	3:1	4:1	5:1	6:1	7:1	8:1	9:1	10:1
V1 (V)	4	2.9	2.7	2.5	2.42	2.35	2.31	2.27	2.24	2.21	2.2
S21 (dB)	− 3.46	− 2.64	− 2.22	− 1.72	− 1.52	− 1.35	− 1.26	− 1.18	− 1.12	− 1.072	− 1.05
S31 (dB)	− 3.47	− 4.51	− 5.32	− 6.74	− 7.57	− 8.45	− 9.03	− 9.67	− 10.20	− 10.77	− 10.97
Dif. (dB)	0.01	1.87	3.1	5.02	6.05	6.93	7.76	8.5	9.08	9.7	9.91

**Table 2:** Important ranges of divided power for second design TWPD at V1 = 4.

Range	1:185	1:267	1:93	1:35	1:17	1:10	1:6	1:4	1:3	1:2	1:1
V2 (V)	1.7	1.8	1.9	2	2.1	2.2	2.31	2.42	2.5	2.7	4
S21 (dB)	− 23.45	− 25.02	− 20.47	− 16.22	− 13.11	− 10.78	− 8.86	− 7.42	− 6.60	− 5.22	− 3.46
S31 (dB)	− 0.775	− 0.757	− 0.77	− 0.825	− 0.93	− 1.082	− 1.31	− 1.57	− 1.77	− 2.28	− 3.47
Dif. (dB)	22.68	24.26	19.7	15.4	12.19	9.71	7.55	5.95	4.83	2.94	0.01

**Table 3:** Properties comparison of two WPDs with other works (MPDR: Maximum power-dividing ratio).

Ref./Type	Harmonic suppression	Symmetric two-sided	Blocking signal	*f*_o_ (GHz)	FBW (%)	IL_min_–IL_max_ (dB)	RL (dB)	MPDR*	FPDR (%)	Size (${\lambda }_{g}\times {\lambda }_{g}$)
^28^/TWPD	–	–	–	1.05	66.7	2.1–5.8	–	1:2.4	37.21	0.25 × 0.02
^29^/TWPD	–	✓	–	2.4	–	0.94–12	20	1:28	88.87	0.29 × 0.13
^30^/TWPD	–	–	–	1	–	–	–	1:100	–	–
^32^/TWPD	✓	✓	–	1.5	58	0.8–1.8	–	1:2	27.78	0.24 × 0.12
^33^/TWPD	✓	–	–	1.09	42–54	0.8–1.3	16–21	1:3	25.64	0.26 × 0.14
First design	✓	✓	✓	2.5	3.6	1.76–18.33	10.4–20.78	1:45	88.39	0.23 × 0.28
Second design	✓	✓	✓	2.52	5.55	0.88–22.15	15.15–18.73	1:134	95.31	0.21 × 0.31

The novelty of the proposed work is divided into the two parts: the novelty of the design and the novelty of the results as follows:

i. Power dividers are designed based on the bandpass filter (BPF) with new structure consisting of high- and low- impedance lines. Power dividers with tunable power-dividing ratio have a circuit consisting of lumped elements. The varactor diode in the lumped elements circuit act as variable capacitor, where its capacitance is depending on the DC voltage. In the proposed power divider structure, the variable capacitors are added to low-impedance line TL1s, which has the greatest capacitive and high coupling effects. As a result, besides a novel structural configurations, another design novelties are controlling of capacitive and coupling effects of low-impedance line. Adding lumped element circuit to low-impedance line with high capacitive and coupling effects have not be seen in the previous works. Previous works^28–33^ just used lumped elements circuit with high inductive effect lines (high-impedance line). Therefore, the proposed design novelty causes the less number of lumped elements.
ii. The novelty of the results or the advantages of the proposed TWPDs in compare with previous works are the wide ranges of the power-dividing ratio (for the first time up to 1:135), harmonic suppression with wide stopbands, symmetric two-sided power dividing, completely blocking signal for both ports or any desired port (for first time). Unlike the previous works, all these advantages are included in the two proposed power dividers. Also beside a feature of power dividing, a complete blocking of the signal at the central frequency for two output ports, is a unique feature, which was not presented in the previous works.

## Conclusion

This paper has presented two tunable Wilkinson power dividers with wide range of power-dividing ratio. The first idea is design of a novel narrow-band BPF based on the equivalent circuit model and LC equivalent circuit using odd/even mode analysis. Then, by investigation of surface current density, it is determined by which part of designed BPF the insertion loss can be controlled at *f*_o_. The TWPDs are designed based on IL control components to create a wide range of power-dividing ratios. The structure of TWPDs are consisted of two designed TBPFs, two lumped element circuits and two DC voltages. The advantages of the proposed TWPDs are included these cases: the wide ranges of power-dividing ratio equal to unequal, compact size, the low number of lumped elements, harmonic suppression with wide stopbands, symmetric two-sided, completely blocking signal and the use of only two DC voltages. Comparison of simulation and measurement results show that there has been a good agreement between the results, and thus confirms the correctness of the designs.

## Data Availability

The calculated results during the current study are available from the corresponding author on reasonable request.
